# Role of Imaging in the Management of Patients with SARS-CoV-2 Lung Involvement Admitted to the Emergency Department: A Systematic Review

**DOI:** 10.3390/diagnostics13111856

**Published:** 2023-05-26

**Authors:** Cesare Maino, Paolo Niccolò Franco, Cammillo Talei Franzesi, Teresa Giandola, Maria Ragusi, Rocco Corso, Davide Ippolito

**Affiliations:** 1 Department of Diagnostic Radiology, IRCCS San Gerardo dei Tintori, Via Pergolesi 33, 20900 Monza, Italy; mainocesare@gmail.com (C.M.); maria.ragusi@gmail.com (M.R.);; 2 School of Medicine, University of Milano Bicocca, Via Cadore 33, 20090 Monza, Italy

**Keywords:** infections, coronavirus, radiography, tomography, X-ray computed, ultrasonography, artificial intelligence, SARS-CoV-2, COVID-19

## Abstract

During the waves of the coronavirus disease (COVID-19) pandemic, emergency departments were overflowing with patients suffering with suspected medical or surgical issues. In these settings, healthcare staff should be able to deal with different medical and surgical scenarios while protecting themselves against the risk of contamination. Various strategies were used to overcome the most critical issues and guarantee quick and efficient diagnostic and therapeutic charts. The use of saliva and nasopharyngeal swab Nucleic Acid Amplification Tests (NAAT) in the diagnosis of COVID-19 was one of the most adopted worldwide. However, NAAT results were slow to report and could sometimes create significant delays in patient management, especially during pandemic peaks. On these bases, radiology has played and continues to play an essential role in detecting COVID-19 patients and solving differential diagnosis between different medical conditions. This systematic review aims to summarize the role of radiology in the management of COVID-19 patients admitted to emergency departments by using chest X-rays (CXR), computed tomography (CT), lung ultrasounds (LUS), and artificial intelligence (AI).

## 1. Introduction

After the initial outbreak in Wuhan in December 2019, the World Health Organization (WHO) reported that, as of 10 May 2023, there have been 765,903,278 confirmed cases of COVID-19, including 6,927,378 deaths (data available on 15 May 2023) [1]. Despite the WHO ending the global emergency status for COVID-19 on 11 May 2023, it is of the utmost importance to analyze what occurred over the previous few years and to underline what we have learned.

Severe Acute Respiratory Syndrome Coronavirus (SARS-CoV-2) infection results in a wide spectrum of clinical features, from totally asymptomatic to severe acute respiratory distress syndrome (ARDS), thus making the diagnostic process challenging [2].

As reported in a recently published meta-analysis, saliva and nasopharyngeal swab Nucleic Acid Amplification Testing (NAAT) reported similar diagnostic accuracies and should be considered the first testing choice in all medical settings [3]. The authors reported a pooled sensitivity and specificity for saliva NAAT of 85.6% (95% CrI = 77.0–92.7%) and 99.1% (95% CrI = 98.0–99.8%), respectively, while the nasopharyngeal swab NAAT showed a sensitivity and specificity of 85.7% (95% CrI = 76.5–93.4%) and 98.9% (95% CrI, 97.4–99.7%), respectively.

In these settings, a combination of clinical, radiological, and laboratory parameters may increase sensibility and specificity values, helping clinicians to maximize available resources and establish the final diagnosis.

Based on its main pathological mechanism, COVID-19 should not be considered as a single disease; the angiotensin converting enzyme (ACE2) receptor is the entrance door for the virus, which can spread and duplicate not only in the lungs (where the ACE2 receptor is over-expressed), but in different tissues and organs.

The most common symptoms are linked to lung involvement, including fever, cough, shortness of breath, fatigue, loss of taste or smell, sore throat, and congestion. Symptoms may appear 2–14 days after exposure and can range from mild to severe [4,5].

To enhance clinical accuracy, the radiological approach can be used to solve the differential diagnosis as soon as possible and to manage patients quickly and correctly. From the radiological point of view, asymptomatic patients may present slight and limited pulmonary opacities [6]. In the first two days from symptom onset, computed tomography (CT) findings are normal in just over 50% of cases [7]. During the first week (early phase), ground-glass opacities (GGOs) are common, and their rapid progression in consolidations is a common finding. During the disease’s progression, GGOs can be associated with reticulation (crazy-paving pattern). Subsequently, at around 2–3 weeks, signs of organized pneumonia can be seen [8].

Even if different strategies were reported as useful to stratify patients according to disease severity, one of the most accepted is the CT-based semiquantitative strategy [9]. The total CT score is measured by the sum of the individual lobar scores and can range from 0 (no involvement) to 25 (maximum involvement).

As the Fleischner Society pointed out [10], many scenarios may occur, thus challenging the healthcare staff to pursue the correct diagnostic path. In this setting, chest imaging, including chest X-ray (CXR) and CT, can be fundamental; this is particularly the case with negative NAAT results but with suggestive clinical symptoms [11].

Regarding the cost-effectiveness of imaging, an interesting study that enrolled a large cohort of consecutive patients with COVID-like symptoms was published in 2022 [12]. The authors demonstrated that ultra-low dose CT was the more cost-effective approach to decrease the length of stay in the emergency department (ED).

On these bases, this systematic review aims to summarize the most important actual evidence regarding the use of the radiological approach in reaching the final diagnosis of patients suspected of SARS-CoV-2 lung involvement admitted to the ED.

## 2. Search Strategy

We systematically searched MEDLINE (PubMed interface) and EMBASE (Elsevier interface) on 21 February 2023 using the following search keywords and relative equations: (1) “COVID-19 AND radiology AND emergency”, (2) “SARS-CoV-2 AND radiology AND emergency”, (3) “SARS-CoV-2 AND computed tomography AND management”, and (4) “SARS-CoV-2 AND X-ray AND management”.

The obtained records were imported into Rayyan (https://www.rayyan.ai (accessed on 21 February 2023) for screening. After the first automatic screening, duplicates were excluded.

Two radiologists, C.M. and P.N.F., with 7 and 5 years of experience, respectively, examined titles, keywords, and abstracts. The reviewers excluded conference abstracts, posters, and conference papers. Published studies not in English were excluded as well. After the manual screening process, the reviewer analyzed the full text of each paper. Moreover, both readers examined all references lists of included papers and added pertinent articles related to the abovementioned topic, as appropriate.

### Included Studies

The literature search identified 7094 publications. Using the Rayyan website, 2129 studies were excluded due to duplication, while 715 were marked as ineligible by keywords selected by the two reviewers.

**Figure 1:**
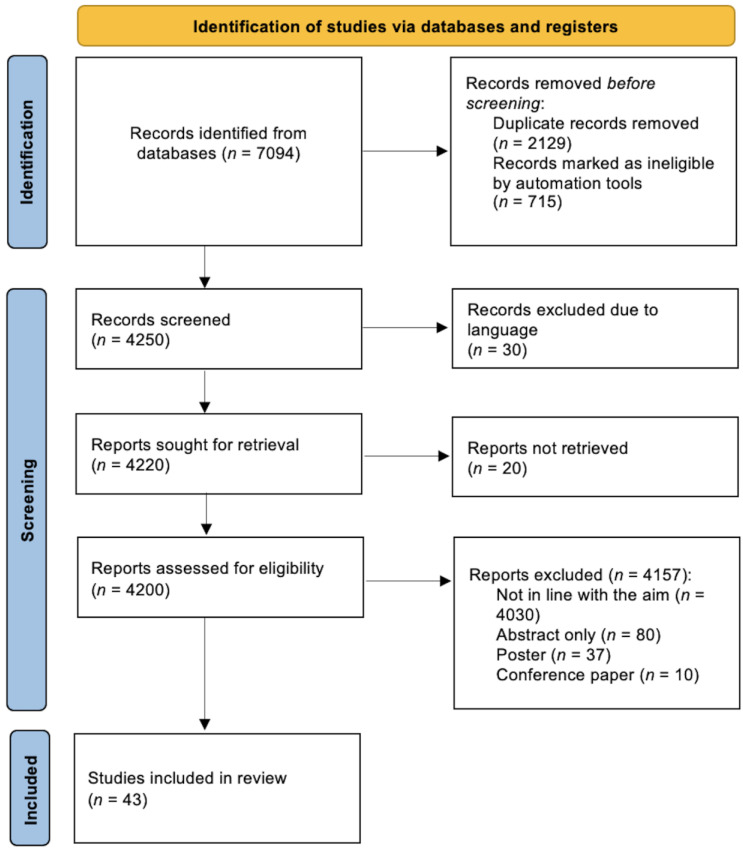
PRISMA flowchart of the study.

Starting with a total of 4250 studies, the reviewers, by reading all titles and abstracts, excluded 4157 papers deemed to be not in line with the aim of this systematic review. The total number of included studies was 43. The PRISMA flowchart of the study is reported in Figure 1.

## 3. Chest X-ray (CXR)

CXR can play a crucial role in the assessment of SARS-CoV-2-related pneumonia in the ED, primarily due to its widespread availability [13,14]. The CXR is economically convenient and can be performed in every setting, with almost no time span, and, eventually, directly at the patient’s bedside [3], reducing the infection’s spread.

Although less sensitive than chest CT, CXR can be considered the first-line imaging technique in patients suspected of SARS-CoV-2 lung involvement [15]. Moreover, to guarantee decontamination and to avoid transmission between patients and the hospital staff, a portable unit should be preferred [16]. In these settings, installing glass between the portable unit and the patients has been proposed [17].

**Figure 2:**
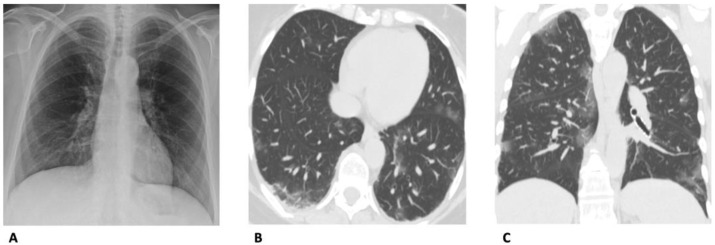
Comparison between CXR and CT findings in a 62-year-old woman with SARS-CoV-2-related pneumonia. (**A**) Anteroposterior chest X-ray was reported negative for lesions of SARS-CoV-2-pneumonia. The subsequent CT in the axial (**B**) and coronal (**C**) planes set with the parenchymal window width, however, showed multiple, bilateral, slight GGOs, especially in the subpleural areas.

From a diagnostic point of view, the most frequent CXR findings are airspace opacities, whether described as consolidation or, less commonly, GGOs (66%), with bilateral (61%) and peripheral (59%) predominance (Figure 2). Pleural effusion is rare (6%) [13].

In the present systematic review, a total of 14 studies were strictly focused on the usefulness of CXR in the management of patients admitted to the ED.

The first step to consider during ED admission is the triage procedure. During the first peak of the pandemic, some authors [18] proposed CXR as a valuable tool to triage patients. The authors suggested that a Likert scale system based on CXR findings can help to solve the differential diagnosis quickly. Thanks to this approach, it is possible to identify patients with a likelihood of SARS-CoV-2 lung involvement and triage them appropriately.

To endorse the usefulness of CXR in the triage process, some authors [19] showed that 65.8% of enrolled patients (*n* = 2225) reported a normal CXR while 34.2% had abnormal findings. The authors used CXR as a screening procedure, and patients with abnormal radiographic findings underwent NAAT. The encouraging finding was that among 52 patients suspected of SARS-CoV-2 pneumonia with normal CXR, only 10 showed a positive NAAT (19%).

Since the first wave of the pandemic, some authors [13] have investigated the role of CXR in the detection of COVID-19 lung involvement in 518 patients admitted to the ED. The authors reported that CXR had a sensitivity and specificity of 57% and 89%, respectively, and that this rate was higher in patients with symptom onset longer than five days.

Other authors [20], by enrolling 117 patients suspected of SARS-CoV-2 pneumonia and hospitalized with lower respiratory tract symptoms, demonstrated that CXR findings were significantly more evident in patients affected with COVID-19 when compared with the non-COVID-19 group (83 vs. 42%, *p* < 0.001). Moreover, the authors reported that CXR abnormalities observed during admission could be considered as predictors for prognosis; patients with abnormal CXR findings reported significantly higher mortality (*p* = 0.0014). In a more extensive study [21], 756 patients were enrolled, of whom 67% with positive NAAT tests showed abnormal CXR.

CXR at admission can be considered as a useful tool not only to triage patients and to solve the differential diagnosis but also to determine patient outcome. A recently published study [22], collecting CXR findings in patients affected with COVID-19 at admission and during the hospitalization, demonstrated that the Radiographic Assessment of Lung Edema (RALE) score can be considered a useful tool to select patients who need hospitalization, with a good reliability between readers. Similarly, some authors [23] aimed to correlate the lung involvement and outcome of patients admitted to the ED. The authors noted that the RALE score, generally used to evaluate pulmonary edema, can be considered as a good prognostic factor in patients with SARS-CoV-2 lung involvement.

Moreover, since 2020, some authors [24], by enrolling 1203 CXRs of 175 patients, aimed to determine the usefulness of CXR in determining admission to intensive care units by using a chest radiography score (CARE). This study reported that CARE has a good accuracy (AUC = 0.736) in detecting patients at risk of intensive care admission, representing a reliable approach to determining lung severity involvement.

Table S1 Summarizes the most important papers regarding the usefulness of CXR.

## 4. Computed Tomography (CT)

Chest CT is considered the reference standard for the evaluation of lung involvement of different pathological entities, primarily due to its intrinsic lung resolution, high sensitivity and specificity, wide availability, and acceptable costs. On the other hand, the most important disadvantages of CT are its reliance on radiation dose exposure, which is to be balanced according to the ALARA (As Low As Reasonably Achievable) principle [25]. Thanks to technological improvements, low-radiation-dose CT images can be obtained by reducing the kV setting or by applying different reconstruction algorithms, including deep learning-based algorithms, to reduce noise and increase spatial resolution [26]. As for other medical aims, low-radiation-dose CT can also be considered helpful in patients with SARS-CoV-2 infection without any significant differences in terms of diagnostic quality when compared with standard CT [27].

In these settings, sub-millisievert chest CT played an essential role during the first wave of the pandemic. In 2020, some authors used low-dose chest CT for the prompt diagnosis of patients admitted to the ED; this CT technique was deemed prompt when compared with the reference standard NAAT. The study highlighted the excellent diagnostic accuracy of CT, with sensitivity, specificity, positive predictive value (PPV), negative predictive value (NPV), and accuracy of 86.7%, 93.6%, 91.1%, 90.3%, and 90.2%, respectively. The overall mean effective radiation dose was 0.56 mSv ± 0.25 [28].

To assess SARS-CoV-2 lung involvement, chest CT should be performed in the unenhanced phase, considering that the primary CT findings are similar to atypical pneumonia. However, the use of contrast media should be strongly recommended in cases where there is the suspicion of pulmonary embolism [29].

CT findings in patients affected with COVID-19 have been widely studied and reported during and after all waves of the pandemic. The typical CT appearance of SARS-CoV-2 lung involvement is bilateral, subpleural GGOs, with lower lobes and posterior predominance (>70%). Less frequently, lung consolidations (50%), linear opacities (40%), interstitial thickening (50%), crazy-paving appearance (35%), pleural thickening (35%), halo sign (35%), bronchiectasis (24%), and nodules (20%) were reported. It has also been reported that pathological findings can be present unilaterally (15%), or with middle and upper lobe involvement (50%) exclusively. Atypical findings, with less than 10% prevalence, should always be considered; these include pleural effusion (5%), nodes enlargement (5%), tree-in-bud appearance (4%), pericardial effusion (3%), and excavated lesions (1%). However, all the above-reported CT findings lack pathological confirmation, as underlined by Kwee [30].

In the present systematic review, a total of 10 studies were strictly focused on the use of CT in the management of patients admitted to the ED.

**Figure 3:**
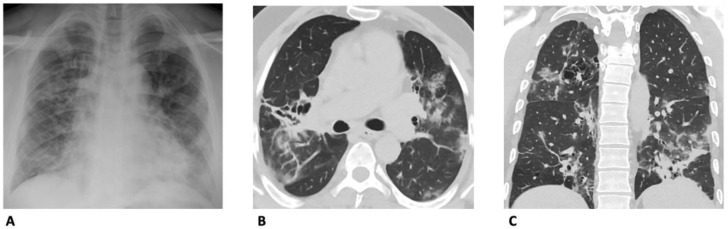
Comparison between chest X-ray and CT findings suggestive of SARS-CoV-2-related pneumonia in a 37-year-old man. (**A**) Chest X-ray shows the presence of multiple confluent mixed alveolar and linear opacities. CT images in the axial (**B**), and in the coronal plane (**C**) with window width and level for the evaluation of lung parenchyma allows to correctly identify the presence of the confluent ground-glass opacities, consolidations, and the additional finding of emphysema associated with bronchiectasis in the right upper lobe and both lower lobes.

As with CXR, chest CT can play an essential role in triaging patients at ED admission. Since the first wave of the pandemic, a prospective, single-center cohort study that enrolled 165 patients aimed to determine the usefulness of CT in the prompt diagnosis of SARS-CoV-2 lung involvement [31]. Using NAAT as the reference standard, the authors reported that low-dose CT had a sensitivity and specificity of 84.6% and 94.7%, respectively. Moreover, the positive and negative likelihood ratios were more than acceptable (16.1 and 0.16, respectively). On these bases, the authors suggested that CT can play a supporting role in the quick diagnosis and management of suspected COVID-19 patients. Similarly, a study published in 2021, enrolling 330 patients, highlighted the usefulness of chest CT in the emergency department and noted its overall importance, especially in regard to the false negative results reported in the initial NAAT (Figure 3) [32].

Despite this, it is of utmost importance to underline that one of the most important limitations of CT is its low availability in low-income countries. Moreover, even if CT is considered a quick procedure in wealthy countries, different geographical areas lack advanced medical equipment, worsening the effort to fight against the COVID-19 pandemic.

**Figure 4:**
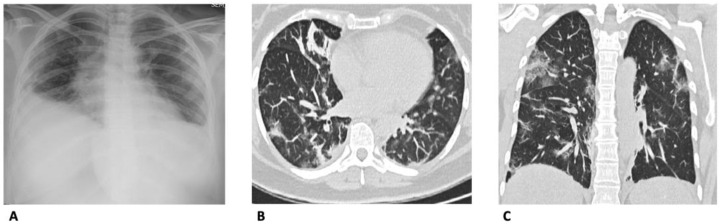
Discordant imaging findings between chest X-ray and CT in a patient with SARS-CoV-2-related pneumonia. Lesions suggestive for SARS-CoV-2-pneumonia were not evident on anteroposterior CXR (**A**). On CT [(**B**) in the axial and (**C**) coronal planes], however, multiple reticulations and GGOs are found in the subpleural areas of the lungs bilaterally.

One of the most common problems in the evaluation of chest CT images is readers’ confidence in the final diagnosis of SARS-CoV-2 lung involvement. In this specific setting, some authors [33] grouped patients into three classes according to subjective probability. In this study, CT specificity and sensitivity were 76% and 99%, respectively, with more than acceptable NPV and PPV (97% and 90%, respectively). With these results, the authors suggested that CT can be considered to be a quick approach for triaging patients who are waiting for the final diagnosis obtained by NAAT (Figure 4).

To better allow communication between clinicians and to develop a standardized reporting system, a COVID-19 reporting and data system (CO-RADS) classification was proposed in 2020 for a use in a moderate to high prevalence setting [34]. The classification is based on the typical findings of SARS-CoV-2 lung involvement, ranging from CO-RADS 1, with normal or non-infection abnormalities, to CO-RADS 5, representing the highest suspicion of COVID-19 infection; CO-RADS 6 is strictly linked to the positive NAAT. To depict the clinical applicability of CO-RADS, in 2022, a large meta-analysis included 24 studies with more than 8000 patients was published [35]. The authors demonstrated that the pooled sensitivity and specificity for CO-RADS ≥ 3 were 89% and 68%, respectively; in addition, a significant increase in specificity for CO-RADS ≥ 4 (84%) with no impact on sensitivity (83%) was reported. When applying CO-RADS to the ED, it is possible to enhance the final diagnosis, as reported in a retrospective study that enrolled 280 patients [36]. The authors endorsed the importance of CO-RADS 5 patients, who had a statistically higher number of positive NAATs when compared to other classes.

As mentioned above, chest CT can be considered the reference standard for the diagnosis of PE, especially in patients admitted to the ED. Considering the direct and indirect damage to the endothelial cells, SARS-CoV-2 can increase the risk of vascular complications, particularly the involvement of pulmonary vessels [37]. During the first waves of the pandemic, some authors reported the usefulness of CT pulmonary angiography (CTPA) for detecting PE in COVID-19 patients [38]. By conducting a retrospective review of CTPA used in their institution, the authors reported that the demand for CTPA in the ED was inappropriate. In fact, according to the collected data, COVID-19 was not considered an independent predictor in the development of PE.

Chest CT at admission can also play a role as a prognostic factor for patients affected by SARS-CoV-2. In a study published in 2021, by enrolling 53 patients and 137 CT scans, the Authors demonstrated good feasibility of the CT scoring system as a predictive factor for clinical severity and its usefulness for clinical treatments [39].

CT images provide plenty of information, particularly when considering a quantitative approach. In 2022, one study [40] aimed to determine if quantitative parameters obtained by COVID-19 chest CT could play a role in predicting respiratory failure. Using commercially available software, high attenuation areas, representing GGOs, were quantified. It has been observed that the amount of GGOs was significantly higher in patients with worse respiratory outcomes. The primary result of the study was that a decision tree composed of clinical and radiological biomarkers had higher accuracy in determining patients’ prognoses.

Table S2 Summarizes the most important papers regarding the usefulness of chest CT.

## 5. Management Strategies

**Figure 5:**
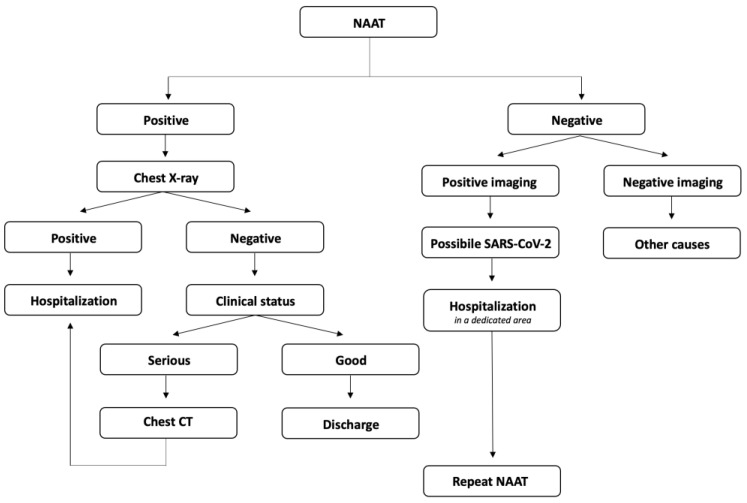
Management flow-chart of patients during ED admittance.

As the SARS-CoV-2 pandemic spread across the globe, the population of patients admitted to the ED became highly heterogeneous [41]. As reported by the Fleischner Society [10], several possible clinical scenarios can occur during ED admittance (Figure 5), and each requires a different approach to imaging when considering the capability to assess the burden of disease [42]. In this setting, chest imaging, including both CXR and CT, can be fundamental, especially in the case of a negative NAAT result and a suggestive clinical history [11,43]. More and more patients were admitted to the ED with atypical symptoms, including neurological or cardiovascular ones, or no symptoms at all. Consequently, imaging was crucial to solve the clinical suspicion.

According to some authors [13,44], most SARS-CoV-2 patients have similar CXR findings, though sensitivity decreased when the onset of symptoms occurred only a few days before ED admission [14,45].

Even if considered part of a disease’s typical pattern [10], GGOs are not always good predictors of hospitalization due to several factors: suboptimal imaging acquisition, an underlying lung or systemic disease, and, finally, readers’ experience [46]. Some authors [14,44] found that the involvement of lower zones can be related to a more severe clinical course; this may be helpful information, in addition to the chest severity scores already implemented in the clinical practice.

When symptoms duration was less than five days from admission, NAATs could return as negative, and imaging could be considered a definite diagnostic criterion if typical SARS-CoV-2-related pulmonary alterations were present. In case of a negative report from CXR but high suspicion of disease, chest CT can be considered complementary in order to demonstrate lung alterations.

Another possible scenario contemplates patients with moderate-to-severe symptoms; despite the symptom onset time, these patients usually had a positive NAAT and highly suggestive CXR, so no other imaging was usually required.

However, in case of two negative NAATs collected within 24 h alongside suspicious clinical and radiological data, patients were considered to be at high risk of SARS-CoV-2 infection, and it became fundamental to obtain the diagnosis through chest CT; this allows for the assessment of GGOs typically present in the early phase.

In patients referred to the ED with serious clinical conditions, after the NAAT was conducted, the positive and typical findings on CXR were usually enough to address patients to the best ventilatory support possible, and no further diagnostic investigations were needed.

Similarly, some authors propose an imaging algorithm for COVID-19 patients. If NAAT was negative, no further imaging was needed. On the other hand, in case of positive NAAT, the patient underwent hospitalization and serial CXR or CT to assess complications. While waiting for the definitive NAAT result, a CXR was used to determine possible lung involvement. If the result was normal, the patient was asked to self-quarantine; if it was abnormal, further dispositions were made according to clinical symptoms [47].

The use of chest CT in the ED allows for better understanding lung involvement and helping to better stratify those patients who need close monitoring. In this setting, even if CT is undoubtedly characterized by higher sensitivity and specificity, performing a CT in the ED presents disadvantages, including a huge burden for the radiology and radiographer’s team and a big challenge for continuous infection control; CXR, on the other hand, is considered more practical as first-line radiological screening [15]. Opinions in this regard pointed towards an overall lower baseline sensitivity of CXR when compared to CT, albeit considering it to be a good alternative tool for SARS-CoV-2-related pneumonia. Due to its higher sensitivity, CT can show abnormalities that are not detectable with a CXR, especially in the less extended and in the lower and peripheral zones [48].

## 6. Lung Ultrasounds (LUS)

In the ED, lung ultrasound (LUS) may provide some advantages over other imaging techniques. First, it has the ability to detect subtle lung alterations in the early stage of COVID-19 infection, even in asymptomatic patients [49]. Moreover, it can also provide an immediate diagnostic response in low-resource settings [50] and without exposing patients to ionizing radiation. Finally, LUS may reduce COVID-19 contamination risk as it can be performed at a patient’s bedside, and the equipment sterilization is fast and straightforward [51,52]. Nevertheless, it should be noted that LUS is a highly operator-dependent tool, and the operator’s experience level could affect the diagnostic accuracy [53].

Typical LUS features of COVID-related pneumonia include three main categories: B-lines, distorted pleural lines, and consolidations [52,54]. B-lines are reverberation artifacts, also defined as “comet tail artifacts”, and are visualized as vertical hyperechoic lines arising from the pleural line and move synchronously with breath. B-lines are not specific to COVID-19 disease since they represent the interstitial involvement of many entities. However, the distribution (bilateral and with an upper anterior-to-lower posterior gradient) may help distinguish COVID-19 pneumonia from other conditions [55]. Furthermore, alterations of normal pleural lines, including discontinuity, fragmentation, and reduced smoothness, are frequently observed in the involved pulmonary zones. Nevertheless, these signs are also unspecific findings since they are commonly observed in acute respiratory distress syndrome (ARDS) and pulmonary fibrosis, as well as in COVID-19 lung involvement. Finally, subpleural pulmonary consolidations can vary from small consolidated areas (patchy, strip, or nodular) to extended consolidations with air bronchograms [56].

In the present systematic review, a total of 11 studies were strictly focused on the use of LUS in the management of patients admitted to the ED.

Different studies proposed LUS as a valuable method for triaging patients with suspected COVID-19 infection in the ED. During the first pandemic wave, some authors proposed an LUS-guided triage system based on integrating patients’ oxygen saturation levels and NAAT with LUS findings. Through this preliminary triage algorithm, patients could be divided into low- and high-risk subjects, allowing identification of those needing hospitalization [57].

Other authors evaluated the possible role of LUS as a triage method in primary care for patients presenting with symptoms compatible with COVID-19 pneumonia. In their retrospective study, the authors compared LUS and CT diagnostic values in the ED setting. LUS showed high sensitivity and NPV (93.3% and 94.1%, respectively) and poor values for specificity, PPV, and accuracy (21.3%, 19.2%, and 33.3%, respectively). On the contrary, chest CT had excellent sensitivity, specificity, NPV, and accuracy (80.0%, 86.7%, 95.6%, and 85.6%, respectively) and a moderate PPV value (54.5%) [58]. More recently, a published study compared LUS and CXR as triage methods. They found similar values of sensitivity (93%) but a lower NPV value (55.6%). Conversely, CXR showed lower values, with a sensitivity of 75% and an NPV of 40%. Consequently, the authors concluded that LUS is an efficient triage method since it can accurately rule out the occurrence of pulmonary involvement in patients with clinical suspicion of COVID infection [59].

A large international multicenter investigation concluded that the combination of patterns and clinical phenotypes (high, mild, or mixed) at presentation can rapidly identify those patients with or without COVID-19 pneumonia at the bedside. The high or intermediate pattern of LUS-likelihood of COVID-19 pneumonia showed an overall sensitivity of 90.2% when identifying patients with positive NAAT, showing higher values in the high (97.1%) and mixed (94.7%) clinical phenotype [60]. Similarly, some authors found that a combination of LUS and clinical findings may increase the identification of false-negative SARS-CoV-2 NAAT results. The LUS-clinical integrated assessment showed a sensitivity and NPV of 94.4% and 95%, respectively, versus 80.4% and 85.2% obtained by NAAT [61]. In a retrospective study, it has been reported that LUS had values of sensitivity, specificity, PNV, and NPV of 92.0%, 64.9%, 88.6%, and 73.3%, respectively, considering two consecutive positive NAAT results as the reference standard [62].

Moreover, a single-center prospective investigation demonstrated that two LUS parameters (the number of involved lung areas and the severity ultrasound score), assessed during the first evaluation in the ED, were significantly associated with a higher risk of intensive care unit admission (*p* = 0.008 and *p* = 0.02, respectively) and death (*p* = 0.01 and *p* = 0.02, respectively) [63]. An analogous study evaluated the ability of an LUS score system, obtained by attributing a score from 0 to 3 to each of 10 lung regions in order to predict different levels of disease severity. The AUC of LUS scores in discriminating severe and critically from moderately ill patients was 0.948, and a LUS score cut-off of 4.5 had a sensitivity of 89.3% and a specificity of 92.9% [64].

Since the first wave of pandemic, several studies have focused on comparing LUS diagnostic performance with other imaging modalities for assessing COVID-19 lung disease. A systematic review published in 2022 identified 51 studies that evaluated the diagnostic accuracy of different imaging modalities in symptomatic patients with suspected COVID-19. It found that CXR, LUS, and chest CT all had moderate sensitivity (80.6%, 86.4%, and 87.9%, respectively), with lower specificity for LUS (54.6%, vs. 71.5% of CXR and 80% of CT) [65]. In a retrospective study on patients admitted to the ED, CT showed better performance for COVID-19 diagnosis when compared to LUS (sensitivity and specificity for CT 90–95% and 43–69% vs. 94–93% and 7–31% for LUS, respectively) [66]. Some authors found out that LUS was more sensitive (88.9%) than CXR (51.9%) in a small cohort of subjects presented to the ED [67]. More recently, LUS detected pulmonary infiltrates in more patients than CXR (81% vs. 63%), with a greater difference among those with suspected (70% vs. 40%) versus confirmed COVID-19 infection (95% vs. 91%) [68].

In conclusion, LUS has proven to be a helpful and immediate diagnostic tool for evaluating lung involvement in lung involvement of COVID-19 in the ED setting. However, the lack of specificity and the high operator dependency represent relevant limitations of this imaging technique.

Table S3 summarizes the most important papers regarding the usefulness of LUS.

## 7. Artificial Intelligence (AI)

Since the first wave of the pandemic, different papers have investigated the potential contribution of AI algorithms in detecting and stratifying patients presenting to the ED with suspected symptoms of SARS-CoV-2 infection in order to allow a rapid triage for further testing or isolation.

In the present systematic review, a total of seven studies were strictly focused on the use of AI applied to diagnostic radiology in the management of patients admitted to the ED.

During the first pandemic surge, some authors trained a deep learning model to segment lung opacities on unenhanced chest CT scans obtained from a large cohort of patients in order to rapidly detect cases with COVID-19 imaging manifestations and correctly triage them. The authors successively validated the model on an external cohort and considered radiologists’ reports as ground truth. The AI-aided triage achieved an AUC of 0.953, with good diagnostic values (sensitivity = 92.3%, specificity = 85.1%; PPV = 79%, and NPV = 94.8%). Interestingly, the model took a significantly lower median time to flag a positive case when compared to the time needed to draft a report (0.55 min vs. 16.21 min) [69]. Other authors used AI models to integrate chest CT findings with clinical symptoms, exposure history, and laboratory testing to more rapidly diagnose COVID-19 lung involvement. The AI system achieved an AUC of 0.92 and showed comparable sensitivity to a senior thoracic radiologist in a population of 905 subjects who underwent NAAT [70].

Similarly, Kataoka et al. developed and validated a machine learning diagnostic model, combining a pre-existing CT-based AI system with clinical features, including blood test data, achieving an AUC of 0.91 [71]. In a published paper, an AI-based system that automatically detects COVID-19 from CT scans has been developed. The deep learning architecture of the proposed method consists of two models: segmentation and classification. The first is used for obtaining lung regions, while the classification model determines COVID-19 positivity in these regions. This system has achieved a sensitivity of 98% and shows promising results in terms of reducing the detection time for physicians in the ED, providing the priority of each CT scan according to the likelihood of positivity [72].

Moreover, AI technologies have been applied for the early diagnosis of COVID-19 by extracting data from CXR. In a 2020 paper, the possible role of AI-aided triaging methods based on imaging was explored. Emergency physicians integrated a previously developed AI algorithm designed to enhance GGOs and consolidations on CXRs into their workflow. Most enrolled physicians agreed that the tool was easy to use in the existing workflow, and 20% reported that the AI algorithm significantly improved clinical decision-making [73]. Some authors developed a deep learning model for fast screening patients by detecting anomalies on CXR images with 96% accuracy for symptomatic subjects and 70.65% for asymptomatic ones.

Similarly, a recent study presented a deep-learning-based model for the rapid detection of COVID-19 on CXR images that could predict SARS-CoV-2 lung involvement with a sensitivity of 97.6% and specificity of 78.6% [74]. Moreover, another AI algorithm was demonstrated as useful for differentiating normal, abnormal, non-COVID-19 pneumonia, and COVID-19 pneumonia applied to a multicenter cohort of thousands CXRs of patients attending EDs. The algorithm achieved an AUC for COVID-19 of 0.86, with a sensitivity of 83%, and a comparable diagnostic performance with those of four board-certified radiologists [75].

In conclusion, the combination of chest imaging and AI can represent a precious tool for fast diagnosing and triaging COVID-19 patients. Nevertheless, greater datasets and external validations are needed to give the currently developed AI algorithms sufficient robustness and reproducibility.

Table S4 summarizes the most important papers regarding the usefulness of AI.

## 8. Conclusions

Nowadays, NAAT is still considered the most used tool to detect patients suspected for COVID-19 infection thanks to its high diagnostic values. Due to the subtle clinical presentation, imaging plays a crucial role in the management of patients attending the Emergency Department with suspected COVID-19 infection. COVID-19 lung disease has a suggestive appearance on CXR, chest CT, and lung ultrasound. According to data reported in the literature, CXR can be used as a first-line imaging, while CT is still considered as the reference standard because of its intrinsic resolution and high diagnostic values. Furthermore, Artificial Intelligence algorithms can represent precious tools for emergency physicians in the rapid assessment of COVID-19 patients.

## Data Availability

Data is contained within the article.

## Supplementary Materials

The following supporting information can be downloaded at: https://www.mdpi.com/article/10.3390/diagnostics13111856/s1, Table S1: Details of collected articles regarding the usefulness of CXR in the management of COVID-19 patients admitted to the ED; Table S2: Details of collected articles regarding the usefulness of chest CT in the management of COVID-19 patients admitted to the ED; Table S3: Details of collected articles regarding the role of LUS in the management of COVID-19 patients admitted to the ED; Table S4: Details of collected articles regarding the role of AI in the management of COVID-19 patients admitted to the ED.
