# Supplementary material for: Role of Imaging in the Management of Patients with SARS-CoV-2 Lung Involvement Admitted to the Emergency Department: A Systematic Review
Source: Diagnostics (Basel). 2023 May 26;13(11):1856. doi: 10.3390/diagnostics13111856 (PMC10252607; doi:10.3390/diagnostics13111856)
Supplement: Supplementary file 1 [file diagnostics-13-01856-s001.zip › diagnostics-2337806-supplementary.pdf]

Supplementary Materials

Table S1. – Details of collected articles regarding the usefulness of CXR in the management of COVID-19 patients admitted to the ED.

Title	Year	Study type	Geographical area*	Main aims	Main findings	PMID
Cost-benefit analysis of portable chest radiography through glass: Initial experience at a tertiary care center during the COVID-19 pandemic	2021	Research article	NA	Cost and benefit of through glass CXR	Increase of overall cost: 9.87 USD per patient with good savings per annum	33875400
Evaluation of Admission Chest X-Ray Findings in Patients With Respiratory Infection During the COVID-19 Pandemic	2021	Research article	E	Prevalence of CXR findings in patients requiring hospitalization	CXR abnormalities are higher in patients with confirmed COVID-19 infection	34692325
Utility of chest radiography on admission for initial triaging of COVID-19 in symptomatic patients	2020	Letter to the Editor	AP	The usefulness of the Likert scale to triage patients	A score higher than 4 had sensitivity and specificity of 76% and 79%	32904474
Role of chest radiography in the management of COVID-19 pneumonia: An overview and correlation with pathophysiologic changes	2021	Research article	AP	Analyze the pattern of CXR findings of suspected or confirmed COVID-19 patients	About 70% of patients had abnormal CXR findings at the admission	33814764
Descriptive analysis of a comparison between lung ultrasound and chest radiography in patients suspected of COVID-19	2021	Research article	E	Compare lung ultrasound and CXR in patients suspected of COVID-19	- Strong disagreement between lung ultrasound and CXR<br>- Lung ultrasound seems to be better than CXR in the detection of COVID-19 lung involvement	33635443

Radiography-based triage for COVID-19 in the Emergency Department in a Spanish cohort of patients	2022	Research article	E	Evaluate the outcome of the triage CXR-based system	- CXR was positive in 62.7%<br>- CXR can be considered an effective tool for triaging patients	35702721
Radiographic patterns on Chest X-ray as a supporting imaging tool in triaging of suspected Corona Virus Disease (COVID) patients	2022	Research article	AP	Evaluate the CXR pattern according to the modified Brixia scoring system	- CXR is useful for triaging patients<br>- CXR can assess disease severity	35991277
Diagnostic Model of COVID-19 Infection Based on the Combination of Clinical Symptoms, Chest Radiography, and Laboratory Test	2022	Research article	AP	The usefulness of CXR in the quick detection of COVID-19 lung involvement	A combination of clinical, laboratory and abnormal findings on CXR can manage the isolation of patients	36156483
Chest Radiograph Severity and Its Association With Outcomes in Subjects With COVID-19 Presenting to the Emergency Department	2022	Research article	NA	Determine the usefulness of CXR in the prognosis of COVID-19 patients	- Radiographic Assessment of Lung Edema (RALE) was used to classify CXRs<br>- RALE reported excellent reliability among readers<br>- Higher RALE values were associated with admission to intensive care units	35473787
Comparison of Chest Ultrasound and Standard X-Ray Imaging in COVID-19 Patients	2020	Research article	E	Compare the usefulness of lung ultrasound and	- Lung ultrasound is useful to	32905446

				CXR in the detection of COVID-19	detect the interstitial syndrome<br>- Lung ultrasound reported higher sensitivity in comparison with CXR	
Chest X-ray in the emergency department during COVID-19 pandemic descending phase in Italy: correlation with patients' outcome	2021	Research article	E	Diagnostic values of CXR at the admission	- CXR showed 83% and 60% of sensitivity and specificity<br>- GGOs and diffuse distribution were independent predictors of COVID-19 diagnosis	33394364
Diagnostic and Prognostic Value of Chest Radiographs for COVID-19 at Presentation	2020	Research article	AP	Diagnostic and prognostic values of CXR	- RALE score failed to identify COVID-19 patients<br>- A high RALE score was associated with a worse prognosis	32970556
Chest X-ray features of SARS-CoV-2 in the emergency department: a multicenter experience from northern Italian hospitals	2020	Research article	E	Diagnosis of COVID-19	- GGOs and interstitial opacities are the main COVID-19 findings in lung involvement<br>- Symptom onset is associated with abnormal CXR findings	32469732

Diagnostic impact of bedside chest X-ray features of 2019 novel coronavirus in the routine admission at the emergency department: case series from Lombardy region	2020	Research article	E	Diagnostic accuracy of CXR	- Sensitivity and specificity were 57% and 89%<br>- Sensitivity was higher for patients with symptom onset > 5 days compared to ≤ 5 days	32485335

SA: South America, N: North America, E: Europe, AP: Asia-Pacific, PMID: PubMed IDentifier.
*according to the first Author's affiliation.

Table S2. – Details of collected articles regarding the usefulness of chest CT in the management of COVID-19 patients admitted to the ED.

Title	Year	Study type	Geographical area*	Main aims	Main findings	PMID
Ruling out COVID-19 by chest CT at emergency admission when prevalence is low: the prospective, observational SCOUT study	2021	Research article	E	CT as a rule-out tool	- Sensitivity and specificity were 84.6% and 94.7%<br>- PPV and NVP were 57.9% and 98.6%<br>- CT can be used as a complementary tool for early COVID-19 exclusion	33435973
Impact of the COVID pandemic on emergency department CT utilization: where do we go from here?	2022	Research article	NA	Analyze the impact of COVID-19 on CT utilization in the ED	Significant increase in CT utilization before and after pandemic periods (35.9 CTs per 100 visits to 41.8 per 100 visits, respectively)	35729442
Chest CT in the emergency department for suspected COVID-19 pneumonia	2021	Research article	E	Diagnostic values of CT	- CT can help classify patients into "highly likely", "likely" and "unlikely" COVID-19<br>- CT specificity, sensitivity, PPV, and NPV were 76%, 99%, 90%, and 97%, respectively	33165767
Computed Tomography Pulmonary Angiography Utilization in the	2022	Research article	NA	- Evaluate the order of CTPA	- CTPA demand increased by 62%	35749621

Emergency Department During the COVID-19 Pandemic				during the pandemic<br>- Evaluate if COVID-19 patients were more affected by PE	- Detection of PE remained stable<br>- COVID-19 is not a predictor of PE at ED admittance	
Radiation dose levels in chest computed tomography scans of coronavirus disease 2019 pneumonia: A survey of 2119 patients in Chongqing, southwest China	2021	Research article	AP	Evaluation of radiation dose exposure in terms of effective dose	- The median effective dose was 4.55 mSv (range 0.11-15.3)<br>- The median number of CT scans for patient was 4<br>- The mean interval between CT scans was 7 days	34397803
Diagnostic performance of low-dose chest CT to detect COVID-19: A Turkish population study	2021	Research article	AP	Diagnostic performance of low-dose CT in the detection of COVID-19 abnormalities	- The sensitivity, specificity, PPV, and NPV of the initial scan were 90.4%, 64.2%, 91.8%, and 60%, respectively<br>- Typical CT findings should be considered more reliable than NAAT	32876571
Coronavirus disease 2019 (COVID-19): chest CT characteristics benefit to early disease recognition and patient classification-a single center experience	2020	Research article	AP	Role of CT in screening patients	- CT abnormalities can occur in the early stage of COVID-19 when NAAT is negative<br>- CT can help with the rapid	32617299

					diagnosis and management of patients	
Quantitative Computed Tomography Parameters in Coronavirus Disease 2019 Patients and Prediction of Respiratory Outcomes Using a Decision Tree	2022	Research article	AP	Role of quantitative CT parameters in classifying COVID-19 patients	- The amount of GGOs is linked to the respiratory outcome<br>- CT quantitative parameters increase accuracy in predicting the outcome	35669915
Accuracy and Reproducibility of Low-Dose Submillisievert Chest CT for the Diagnosis of COVID-19	2020	Research article	E	Evaluate the accuracy of low-dose CT in the diagnosis of COVID-19	- Excellent sensitivity, specificity, PPV, NPV, and accuracy (86.7%, 93.6%, 91.1%, 90.3%, and 90.2%)<br>- CT abnormal findings increase the likelihood of disease from 43.2% to 91.4%	33778576
Comparing the sensitivity and specificity of lung CT-scan with RT-PCR for diagnosis of COVID-19	2022	Research article	AP	Comparison between CT and NAAT in the diagnosis of COVID-19	- Sensitivity, specificity, PPV, and NPV for CT were 94.5%, 24.7%, 40.7, and 89.1%<br>- CT has a better diagnostic value in comparison with NAAT	35726406

SA: South America, N: North America, E: Europe, AP: Asia-Pacific, PMID: PubMed IDentifier.
*according to the first Author's affiliation.

Table S3. – Details of collected articles regarding the role of LUS in the management of COVID-19 patients admitted to the ED.

Title	Year	Study type	Geographical area*	Main aims	Main findings	PMID
Feasibility of using point-of-care lung ultrasound for early triage of COVID-19 patients in the emergency room	2020	Research article	E	Feasibility of LUS for early triage patients with suspected COVID-19 infection	LUS may provide an early ED triage method for evaluating possible COVID-19 infections. Due to limited specificity, positive LUS findings should be confirmed with NAAT or CT	32910323
Lung Ultrasound as a Triage Method in Primary Care for Patients with Suspected SARS-CoV-2 Pneumonia	2022	Research article	E	Usefulness of LUS to triage patients with suspected COVID-19 infection	Due to its high sensitivity and NPV, LUS is useful as a triage tool for patients with suspected COVID pneumonia	36362647
Lung ultrasound for the early diagnosis of COVID-19 pneumonia: an international multicenter study	2021	Multicenter observational research article	E, N	Evaluate the application diagnostic approach based on a combination of LUS findings with patient's symptoms and clinical history in suspected patients	Higher patterns of LUS likelihood of COVID-19 pneumonia showed a high overall sensitivity in identifying patients with positive NAAT, particularly in those with severe clinical symptoms	33743018
Lung Ultrasonography for the Diagnosis of SARS-CoV-2 Pneumonia in the Emergency Department	2020	Research article	E	Evaluate the sensitivity of an integrated LUS-clinical evaluation approach in the ED	A LUS-clinical integrated assessment showed higher sensitivity and specificity than NAAT	33461884

				comparing it with NAAT		
The role of lung ultrasound as a frontline diagnostic tool in the era of COVID-19 outbreak	2020	Research article	E	Assess the diagnostic accuracy of LUS for COVID-19 pneumonia in a cohort of symptomatic patients admitted to the ED	LUS had good values of sensitivity and PNV and slightly lower values of specify and NPV	33090353
Lung ultrasound findings are associated with mortality and need for intensive care admission in covid-19 patients evaluated in the emergency department	2020	Research article	E	Evaluate the ability of LUS to predict mortality and intensive care unit admission of COVID-19 patients at the time of ED admission	The number of involved lung areas and the LUS-based severity score were significantly associated with a higher risk of intensive care unit admission and death	32798003
Lung ultrasound: a valuable tool for assessing COVID-19 patients with different severity	2022	Research article	A	Correlate a LUS-based score system with disease severity	LUS scores were capable of discriminating severely and critically ill from moderately ill patients (AUC = 0.948)	34935689
What is the diagnostic accuracy of chest radiography, ultrasound, and computed tomography for COVID-19?	2022	Systematic review	N	Compare diagnostic values CXR, LUS, and CT for COVID-19 diagnosis	CXR, LUS, and chest CT showed all moderate sensitivity with the lowest specificity for LUS	34353652
Comparison of admission chest computed tomography and lung ultrasound performance for diagnosis of COVID-19 pneumonia in	2020	Research article	E	Compare diagnostic performance of admission CT and LUS	- Admission CT showed better accuracy than LUS for COVID-19 diagnosis	33091835

populations with different disease prevalence				for the diagnosis of COVID-19	- LUS had high but low specificity	
Point-of-care Lung Ultrasound Is More Sensitive than Chest Radiograph for Evaluation of COVID-19	2020	Research article	N	Compare diagnostic performance of LUS and CXR for the diagnosis of COVID-19	LUS was more sensitive than CXR	32726240
Comparison of Lung Ultrasound versus Chest X-ray for Detection of Pulmonary Infiltrates in COVID-19	2021	Research article	E	Assess the correlation between LUS and CXR for detecting lung infiltrates in COVID-19	LUS detected pulmonary infiltrates more often than CXR	33671699

SA: South America, N: North America, E: Europe, A: Asia-Pacific, Af: Africa, PMID: PubMed IDentifier.
*according to the first Author's affiliation.

Table S4. – Details of collected articles regarding the role of AI in the management of COVID-19 patients admitted to the ED.

Title	Year	Study type	Geographical area*	Main aims	Main findings	PMID
Deep learning-based triage and analysis of lesion burden for COVID-19: a retrospective study with external validation	2020	Research article	A	Develop a deep learning algorithm based on chest CT imaging for rapid COVID-19 triaging	In an external validation set, the AI triage algorithm achieved an area under the curve of 0.953 and a median time of 0.55 min to alert a positive case	32984796
Artificial intelligence–enabled rapid diagnosis of patients with COVID-19	2020	Research article	N, A	Test AI algorithms to integrate chest CT findings with clinical symptoms, exposure history and laboratory testing to rapidly diagnose COVID-19 patients	The AI model achieved an AUC of 0.92 and showed comparable sensitivity to a senior thoracic radiologist	32427924
Integrated model for COVID-19 diagnosis based on computed tomography artificial intelligence, and clinical features: a multicenter cohort study	2020	Research article	A	Test a machine learning diagnostic model based on CT imaging and clinical features	The model achieved an AUC of 0.91	35284557
AI-assisted CT imaging analysis for COVID-19 screening: Building and deploying a medical AI system	2021	Research article	A	Propose an AI-based system for automatically detect COVID-19 from CT scan images	- The system achieved a sensitivity of 0.98<br>- The system reduced physicians detection time	33199977

Deployment of artificial intelligence for radiographic diagnosis of COVID-19 pneumonia in the emergency department	2020	Research article	N	Test the possible role of an AI algorithm based on CXR in the ED workflow	In the 20% of cases the AI algorithm impacted emergency physician clinical decision-making	33392549
Application of deep learning for fast detection of COVID-19 in X-Rays using nCOVnet	2020	Research article	A	Propose a deep learning neural network-based method for fast screening COVID-19 patient by analyzing CXR	The model showed a sensitivity of 97.62% and specificity of 78.57%	32536759
Development and prospective validation of COVID-19 chest X-ray screening model for patients attending emergency departments	2021	Research article	E	Propose an AI algorithm to differentiate normal, abnormal, non-COVID-19 pneumonia, and COVID-19 pneumonia on CXRs	- The algorithm achieved an AUC for COVID-19 of 0.86, with a sensitivity of 83%<br>- The diagnostic performance was comparable with that of four board-certified radiologists	34650190

SA: South America, N: North America, E: Europe, A: Asia-Pacific, Af: Africa, PMID: PubMed IDentifier.
*according to the first Author's affiliation.
